# Implications of using administrative healthcare data to identify risk of motor vehicle crash-related injury: the importance of distinguishing crash from crash-related injury

**DOI:** 10.1186/s40621-024-00523-3

**Published:** 2024-08-12

**Authors:** Nina R. Joyce, Leah R. Lombardi, Melissa R. Pfeiffer, Allison E. Curry, Seth A. Margolis, Brian R. Ott, Andrew R. Zullo

**Affiliations:** 1 Department of Epidemiology, Brown University School of Public Health, 121 South Main St., Box G-121-S2, Providence, RI 02192 USA; 2 Center for Gerontology and Health Care Research, Brown University School of Public Health, Providence, RI USA; 3 Center for Injury Research and Prevention, Children’s Hospital of Philadelphia, Philadelphia, PA USA; 4 Department of General Pediatrics, University of Pennsylvania Perelman School of Medicine, Philadelphia, PA USA; 5 Department of Psychiatry, Rhode Island Hospital, Providence, RI USA; 6 Warren Alpert Medical School of Brown University, Providence, RI USA

**Keywords:** Motor vehicle crash, Older driver, Medicare, Injury, Crash-related injury, Police reported crash

## Abstract

**Background:**

Administrative healthcare databases, such as Medicare, are increasingly used to identify groups at risk of a crash. However, they only contain information on crash-related injuries, not all crashes. If the driver characteristics associated with crash and crash-related injury differ, conflating the two may result in ineffective or imprecise policy interventions.

**Methods:**

We linked 10 years (2008–2017) of Medicare claims to New Jersey police crash reports to compare the demographics, clinical diagnoses, and prescription drug dispensings for crash-involved drivers ≥ 68 years with a police-reported crash to those with a claim for a crash-related injury. We calculated standardized mean differences to compare characteristics between groups.

**Results:**

Crash-involved drivers with a Medicare claim for an injury were more likely than those with a police-reported crash to be female (62.4% vs. 51.8%, standardized mean difference [SMD] = 0.30), had more clinical diagnoses including Alzheimer’s disease and related dementias (13.0% vs. 9.2%, SMD = 0.20) and rheumatoid arthritis/osteoarthritis (69.5% vs 61.4%, SMD = 0.20), and a higher rate of dispensing for opioids (33.8% vs 27.6%, SMD = 0.18) and antiepileptics (12.9% vs 9.6%, SMD = 0.14) prior to the crash. Despite documented inconsistencies in coding practices, findings were robust when restricted to claims indicating the injured party was the driver or was left unspecified.

**Conclusions:**

To identify effective mechanisms for reducing morbidity and mortality from crashes, researchers should consider augmenting administrative datasets with information from police crash reports, and vice versa. When those data are not available, we caution researchers and policymakers against the tendency to conflate crash and crash-related injury when interpreting their findings.

**Supplementary Information:**

The online version contains supplementary material available at 10.1186/s40621-024-00523-3.

## Background

Although motor vehicle crashes are a leading cause of fatal and non-fatal injury in the United States, they are still relatively rare events. (CDC 2019) Thus, it can be logistically challenging (and prohibitively expensive) to prospectively collect information from a large enough sample of drivers to study clinical and pharmacological causes of motor vehicle crashes with sufficient statistical precision. Instead, investigators often rely on databases constructed for administrative purposes, such as hospital discharge data or Medicare insurance claims (“administrative healthcare data”). (Carlson et al. 2016; Ferdinand et al. 2019; Agimi et al. 2018; Leonard et al. 2020; DiMaggio et al. 2021; Ferdinand et al. 2015) With records for millions of people over multiple years, these data usually include information on drivers with crash-related injuries requiring medical care in addition to many clinical exposures of interest, such as the use of prescription drugs and driving-relevant medical conditions (e.g., epilepsy, glaucoma, dementia).

A limitation of administrative healthcare data, however, is that they only contain information on crash-related injuries, not all drivers involved in a crash (“crash-involved drivers”). Without additional information on crashes that did not result in an injury, investigators using administrative healthcare data can only estimate the effect of an exposure on crash-related injury and cannot estimate the effect on the risk of the crash itself. Yet, as the use of administrative healthcare data in traffic safety research has grown, investigators are increasingly failing to distinguish crash from crash-related injury as an outcome. Studies of crash-related injury often motivate the analysis by citing prior literature on the risk of a crash, (Carlson et al. 2016; Agimi et al. 2018) while others identify a crash as the primary outcome of interest, but only include data on crashes that result in an injury. (Redelmeier et al. 2015; Asbridge et al. 2021; Brubacher et al. 2019; Gibson et al. 2009)

There is nothing inherently wrong with limiting an analysis to crash-related injuries. When the goal is to develop interventions for high-risk groups of drivers, however, it is important to distinguish characteristics associated with crash from those associated with crash-related injury. For example, obesity is associated with a greater risk of injury once involved in a crash, (Zhu et al. 2006; Viano et al. 2008) but no research has suggested that obesity is associated with a greater risk of the crash itself. Conversely, numerous studies have suggested that older drivers with dementia are at increased risk of a crash, (Ott and Daiello 2010; Brown and Ott 2004) but there is no evidence that dementia is associated with a greater risk of injury once involved in a crash aside from the increased risk for injury or fatality seen for older drivers in general. Thus, while both groups may have higher rates of crash-related injury and thus be considered “high risk,” the mechanism by which their risk is elevated, and therefore the effective intervention to reduce their risk, is materially different.

If the driver characteristics associated with crash and crash-related injury differ, then the growing tendency to conflate the two may result in interventions targeted at the wrong groups of drivers or policies with unintended consequences. In this paper we use a novel data source, Medicare insurance claims linked to crash-involved drivers in New Jersey over a 10-year period–to compare the characteristics of crash-involved drivers with a Medicare claim for a crash-related injury to drivers involved in a police-reported crash irrespective of injury. We show that, across many clinical and pharmacological measures of interest, crash-involved older drivers with a claim for a crash-related injury differ significantly from crash-involved drivers with a police crash report and discuss the implications for analyses using administrative healthcare data to study crash and crash-related injury.

## Methods

*Data sources and linkage:* Data for this study came from the New Jersey Safety and Health Outcomes (NJ-SHO) data warehouse and Medicare claims data for the years 2007 through 2017. Medicare is a federal health insurance program covering inpatient care, outpatient care, and prescription drugs that is available to all United States residents 65 and older, or those with a disability, End Stage Renal Disease (ESRD), or Amyotrophic Lateral Sclerosis (ALS; also known as Lou Gehrig’s disease). Because there are no income requirements, 99% of US adults 65 and older receive health insurance through the Medicare program, though approximately 40% supplement it with additional private insurance. (Lindstrom et al. 2017) The NJ-SHO data warehouse is a repository of various statewide administrative databases linked via probabilistic and deterministic algorithms; a full description of the development and validation of the NJ-SHO are available in a prior paper. (Curry et al. 2021) For the purposes of these analyses, we used the (1) New Jersey driver licensing database, which contains the full licensing record for any individual licensed in New Jersey from January 2004 through December 2018 and (2) crash database, which includes detailed information on all motor vehicle crashes that occur in the state of New Jersey for which there is a police crash investigation report (a “police report”) from 2004 through 2017. A crash is reportable in New Jersey if it results in an injury or more than $500 in damage. (Rutgers University Police Technical Assistance Program) The crash and licensing databases are linked at the level of the individual such that each individual in the licensing database is linked to every crash in which they are identified on the police report.

The Medicare enrollment and fee-for-service claims data include the Medicare Master Beneficiary Summary File (MBSF), MedPAR inpatient claims, outpatient (Part A institutional) claims, and Carrier (Part B professional provider) claims. The Medicare data also contain the Chronic Condition Warehouse (CCW), which combines data across the Medicare files to create indicators of chronic diseases and the first date a beneficiary was diagnosed. Individuals within the NJ-SHO licensing or crash database were linked with Medicare data at the level of the individual using a strict matching algorithm that included last name, birthdate, sex, and zip code. The final NJ-SHO Medicare database (the “NJ-SHO-Medicare bridge”), includes Medicare beneficiaries who had either a New Jersey license, were the driver in a police-reported crash, or both, from 2004 through 2017. The final data set for our analyses includes crashes and claims in 2008 through 2017–the years in which all Medicare datasets were available and allowing for a one-year look-back period prior to the crash to identify clinical diagnoses and prescription medication use (see *Defining crash and crash-related injury in the data* below).

*Study population and analytic sample:* The target population for this study consists of older adults with a valid New Jersey license (i.e., not suspended or expired) from 2008 through 2017 who were residents of New Jersey. Because our focus is on characteristics associated with traffic safety outcomes (crash or crash-related injury), our analytic sample is limited to police-reported crashes and Medicare claims for a crash-related injury in which a member of our target population could have been the driver (i.e., was a resident of New Jersey with a valid New Jersey license).

To identify the analytic sample in the NJ-SHO-Medicare bridge, we included (1) all crash-involved drivers for which there was a New Jersey police crash investigation indicating the driver was 68 years or older and had a valid New Jersey license on the day of the crash and (2) all adults ages 68 years or older with an inpatient or emergency department claim for a crash-related injury as defined by an external cause of injury code (“E-code”) indicating a crash-related injury (Table 1). External cause of injury codes for a crash allow the coder to specify the injured person’s role in the crash (e.g., driver, passenger, pedestrian). However, these designations are notoriously inaccurate. (Bowman and Aitken 2011) Thus, we conducted all analyses using two different sets of criteria to define a claim for a crash-related injury. First, we included all eligible claims for a crash-related injury irrespective of the injured party’s designated role in the crash. Although this definition undoubtedly includes a number of non-drivers, it reflects the definition found in most studies using administrative healthcare data. (Carlson et al. 2016; Ferdinand et al. 2019; Ferdinand et al. 2015; Parreco et al. 2018) Second, we limited eligible Medicare claims to those in which the injured party was designated the driver or was left unspecified.

**Table 1:** External cause of injury codes to identify motor vehicle crash related injuries in Medicare claims

External cause of injury code	Description	Code indicating that the injured party was the driver or was unspecified
*ICD-9*		
E810	Motor vehicle traffic accident involving collision with train	E810.0, E810.2, E810.9
E811	Motor vehicle traffic accident involving re-entrant collision with another motor vehicle	E811.0, E811.2, E811.9
E812	Other motor vehicle traffic accident involving collision with motor vehicle	E812.0, E812.2, E812.9
E813	Motor vehicle traffic accident involving collision with other vehicle	E813.0, E813.2, E813.9
E814	Motor vehicle traffic accident involving collision with pedestrian	E814.0, E814.2, E814.9
E815	Other motor vehicle traffic accident involving collision on the highway	E815.0, E815.2, E815.9
E816	Motor vehicle traffic accident due to loss of control without collision on the highway	E816.0, E816.2, E816.9
E817	Noncollision motor vehicle traffic accident while boarding or alighting	E817.0, E817.2, E817.9
E818	Other noncollision motor vehicle traffic accident	E818.0, E818.2, E818.9
E819	Motor vehicle traffic accident of unspecified nature	E819.0, E819.2, E819.9
*ICD-10*		
V02–V04 (.1 or .9), V09.2, V09.3	Pedestrian injured in collision with vehicle	N/A
V12–V14 (.3, .4, .5, .9), V19.4–V19.6, V19.9	Pedal cycler injured in collision with a vehicle	N/A
V20–V28 (.3, .4, .5, .9), V29.4–V29.9	Motorcycle rider injured in collision with a vehicle	V20-V28 (.3, .4, .9), V29 (.4, .6, .8, .9)
V30–V79 (.4 –.9)	Occupant of a three-wheeled motor vehicle, car, pick-up truck, van, or heavy transport vehicle injured in collision with a vehicle	V30-V38 (.4, .5, .9), V40-V48 (.4, .5, .9), V50-V58 (.4, .5, .9), V60-6 (.4, .5, .9), V70-V78 (.4, .5, .9); V39, V49, V59, V69, V79 (.4, .6, .8, .9)
V80.3–V80.5, V81.1, V82.1	Animal rider or occupant of animal-drawn vehicle injured in collision with vehicle	N/A
V83–V86 (.0-.3)	Occupant of a specialty vehicle injured in a collision with a vehicle	N/A
V87.0- V87.8	Traffic accident of specified type but victim’s mode of transport unknown	V87.0-V87.8
V89.2	Person injured in unspecified motor-vehicle accident, traffic	V89.2
Y32	Crashing of motor vehicle, undetermined intent	Y32

ICD = International cassification of disease

For both the police-reported crashes and the Medicare crash-related injuries we required a minimum 12-months of continuous enrollment in Medicare fee-for-service Parts A, B, and D prior to the index date, which was the crash date for police-reported crashes and the admission or visit date for inpatient or emergency department visits. For police-reported crashes we required an additional week of continuous enrollment, at minimum, following the index date to ensure we were able to identify all Medicare claims for an injury related to the crash. Additionally, we required all study members to be a resident of New Jersey as indicated on the Medicare enrollment file in the calendar year that the crash occurred. The home address is updated annually during the enrollment period, which occurs from January 1st through March 31st of each calendar year. Although all adults 65 years and older are eligible for Medicare, we restricted our analysis to individuals 68 and older because we obtained information on clinical covariates prior to the crash from the CCW, which required beneficiaries to be enrolled for at least three years in order to identify some clinical diagnoses.

*Defining crash and crash-related injury in the data:* By definition, all individuals with a crash-related injury must have been involved in a crash. However, there is a subset of crash-related injuries in Medicare claims for which there is no corresponding police report indicating a crash occurred. There are a number of potential reasons for this, including that the crash occurred in New Jersey but was not reported to the police or the crash occurred outside of New Jersey. Thus, to limit our sample to crash-related injuries that *could* be associated with a New Jersey police crash report, we only included crash-related injuries that did not link to a police report if the treating facility on the Medicare claim was in the state of New Jersey as a proxy measure for the crash occurring in New Jersey.

*Driver characteristics:* Age, sex, and race/ethnicity were obtained from the MBSF, while all clinical conditions were determined from the CCW. We included conditions that have been identified as potentially driving-relevant conditions by the American Geriatrics Society and the National Highway Traffic Safety Administration (supplementary Table 3). (American Geriatrics Society A. Pomidor. 2016) We created binary indicators of whether someone had ever been diagnosed prior to the date of the crash based on the first date of diagnosis in the CCW.

All prescription drug information came from the Medicare Part D files. We included medications that prior literature has suggested may (positively or negatively) impact the risk of a crash or a crash-related injury, with one exception—we did not include benzodiazepines because Medicare Part D did not cover them until 2013. (Sundelin et al. 2018; Hansen et al. 2015; Rapoport et al. 2011; Monárrez-Espino et al. 2013; Amanda Hetland 2014; Monárrez-Espino et al. 2014) Because we could not obtain information on the nature of the crash for crash-related drivers with a Medicare claim for a crash-related injury *only* we did not include any crash characteristics (e.g., a left turn, speeding) in our comparison.

*Statistical analyses:* We compared all crash-involved drivers with a police reported-crash to those with an eligible Medicare claim for a crash-related injury. We calculated descriptive statistics and estimated the standardized mean difference for each of the binary covariates and the Mahalanobis difference for the categorical variable of race/ethnicity. The standardized mean difference computes a mean difference between two groups in standardized deviation units, thereby permitting comparisons with other effects sizes measured in different units. We excluded the overlap between the groups (i.e., crash-involved drivers with both a police report and a Medicare claim for a crash-related injury) when calculating the pooled standard deviation for the calculation of the standardized mean difference and considered a difference of greater than 0.1, which translates into a difference of less than 10% of a standard deviation, to indicate no meaningful difference between groups. We based this cutoff on the standard practice in the medical literature, which is a conservative approach given the original formulation considered a “trivial” difference be less than 0.2. (Schober et al. 2021; Cohen 2013) Because there is overlap between the groups, any differences we observe are due to the distributions of the characteristics in the non-overlapping crash-involved drivers (i.e., those with a police report *only* or a Medicare claim for a crash-related injury *only*). In our primary analyses, however, we chose to compare the overlapping groups because this is how the outcomes are operationalized in research–investigators have access to either the claims or the police reports, but rarely both. In addition, we compared driver characteristics across the three mutually exclusive groups (police report only, Medicare claim for a crash-related injury only, both a police report and a Medicare claim) and provide the *p* value for the chi-square test statistic in supplementary Tables 1 and 2.

**Table 2:** Characteristics of crash-involved drivers by source(s) used to identify the crash (all claims)

	Police reported crash	Medicare claim for a crash-related injury
N (% of all crash-involved individuals)^a^	116,465 (95.6%)	12,301 (10.1%)
*Demographics*		
Age (mean/SD)	76.5 (6.3)	77.2 (6.5)
*Sex*		
Male	56,088 (48.2%)	4,630 (37.6%)
Female	60,377 (51.8%)	7,671 (62.4%)
*Race/ethnicity*		
Black/African American	7,947 (6.8%)	1,064 (8.6%)
White	101,182 (86.9%)	10,575 (86.0%)
Asian	2,502 (2.1%)	239 (1.9%)
Hispanic	1,483 (1.3%)	159 (1.3%)
Unknown/Other	3,351 (2.9%)	264 (2.1%)
*Clinical conditions diagnosed prior to the crash*		
Acute myocardial infarction	5,408 (4.6%)	692 (5.6%)
Alzheimer’s disease and related dementias	10,679 (9.2%)	1,605 (13.0%)
Anxiety disorders	19,766 (17.0%)	2,813 (22.9%)
Cataracts	86,362 (74.2%)	9,603 (78.1%)
Chronic kidney disease	28,244 (24.3%)	3,459 (28.1%)
Chronic obstructive pulmonary disease	29,686 (25.5%)	3,882 (31.6%)
Congestive heart failure	34,840 (29.9%)	4,379 (35.6%)
Diabetes	55,679 (47.8%)	6,321 (51.4%)
Epilepsy	2,077 (1.8%)	339 (2.8%)
Glaucoma	37,294 (32.0%)	4,246 (34.5%)
Hypertension	101,106 (86.8%)	11,085 (90.1%)
Ischemic heart disease	71,698 (61.6%)	8,204 (66.7%)
Migraine and other chronic headache	4,352 (3.7%)	643 (5.2%)
Mobility impairments	2,608 (2.2%)	404 (3.3%)
Peripheral vascular disease	35,171 (30.2%)	4,381 (35.6%)
Rheumatoid/Osteoarthritis	71,531 (61.4%)	8,546 (69.5%)
Schizophrenia and other psychotic disorders	1,575 (1.4%)	238 (1.9%)
Sensory—blindness and visual impairment	245 (0.2%)	40 (0.3%)
Sensory—deafness and hearing impairment	19,516 (16.8%)	2,302 (18.7%)
Stroke or trans ischemic attack	16,929 (14.5%)	2,282 (18.6%)
*Prescription drug dispensing in the 12 months prior to the crash*		
Selective serotonin reuptake inhibitors	15,164 (13.0%)	1,913 (15.6%)
Serotonin norepinephrine reuptake inhibitors	4,203 (3.6%)	534 (4.3%)
Tricyclic antidepressants	2,585 (2.2%)	320 (2.6%)
Antiepileptics	11,193 (9.6%)	1,584 (12.9%)
Anticholinergics	21,170 (18.2%)	2,623 (21.3%)
Antihypertensives	88,193 (75.7%)	9,674 (78.6%)
Antihistamines	10,382 (8.9%)	1,235 (10.0%)
Bisphosphonates	7,840 (6.7%)	933 (7.6%)
Opioid analgesics	32,095 (27.6%)	4,161 (33.8%)
Non-benzodiazepine hypnotics	8,988 (7.7%)	1,056 (8.6%)

^a^Percentages add up to more than 100 because some crash-involved individuals have both a police report and a Medicare claim for a crash-related injury

SD = standard deviation

Note that all claims are included irrespective of the injured party’s designated role in the crash,

## Results

**Fig. 1:**
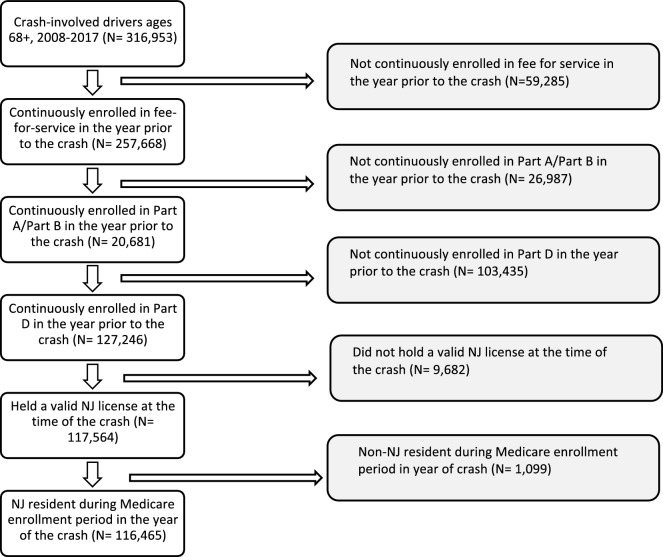
Study cohort flow chart, police-reported crashes

**Fig. 2:**
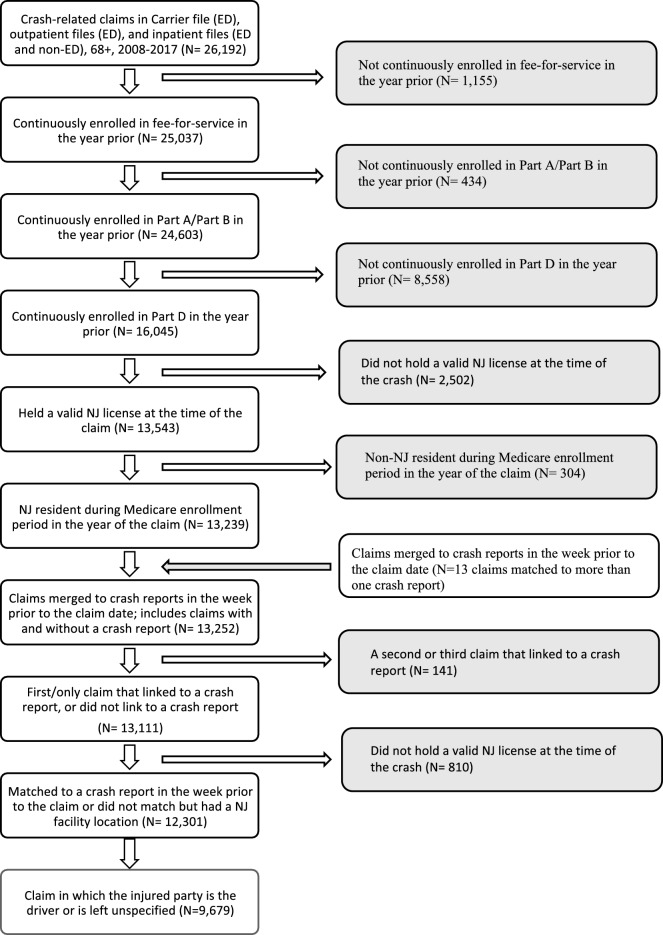
Study cohort flow chart, Medicare claims for a crash-related injury

*Analytic sample:* There were a total of 316,953 crash-involved drivers 68 years and older in the NJ-SHO-Medicare bridge with a police report in the state of New Jersey from 2008 through 2017, of whom 116,465 (36.7%) met eligibility criteria for the analytic sample (Fig. 1). Across the carrier file, outpatient files, and inpatient files there were a total of 26,192 unique Medicare claims with an external cause of injury code for a crash-related injury from 2008 through 2017. When all claims were included irrespective of the injured party’s designated role in the crash, 12,301 (47.0%) met eligibility criteria for inclusion in the analytic sample. When claims were limited to only those where the injured party was designated the driver or left unspecified 9,679 (37.0%) met the eligibility criteria for inclusion (Fig. 2).

After combining crash-involved individuals from both police reports and Medicare claims irrespective of the injured party’s designated role in the crash, there were a total of 121,867 unique crash-involved drivers, of whom 109,566 (89.9%) had only a police report, 5402 (4.4%) had only a Medicare claim for a crash-related injury, and 6899 (5.7%) had both a police report and a claim within seven days of the crash date (supplementary Table 1). Of the 5,402 individuals with only a Medicare claim for a crash-related injury, 1274 (24% of crashes with only a Medicare claim and 1.0% of all crashes) were classified as “unspecified” on the claim while 1629 (30% of crashes with only a Medicare claims and 1.3% of all crashes) were designated as the driver and 2499 (46% of crashes with only a Medicare claim or 2.1% of all crashes) were classified as something else. Thus, the role of the driver could have been misclassified in at most 3.1% of crashes.

When Medicare claims were limited to those in which the injured party was designated the driver or left unspecified, there were a total of 119,245 unique crash-involved drivers, of whom 109,566 (91.9%) had only a police report, 2,903 (2.4%) had only a Medicare claim for a crash-related injury, and 6776 (5.7%) had both a police report and a claim within seven days of the crash date (supplementary Table 2). Of the 2,903 drivers with only a Medicare claim for a crash-related injury, the 1,274 crashes where the driver as left unspecified made up 44% of crashes with only a Medicare claim or 1.1% of all crashes.

**Fig. 3:**
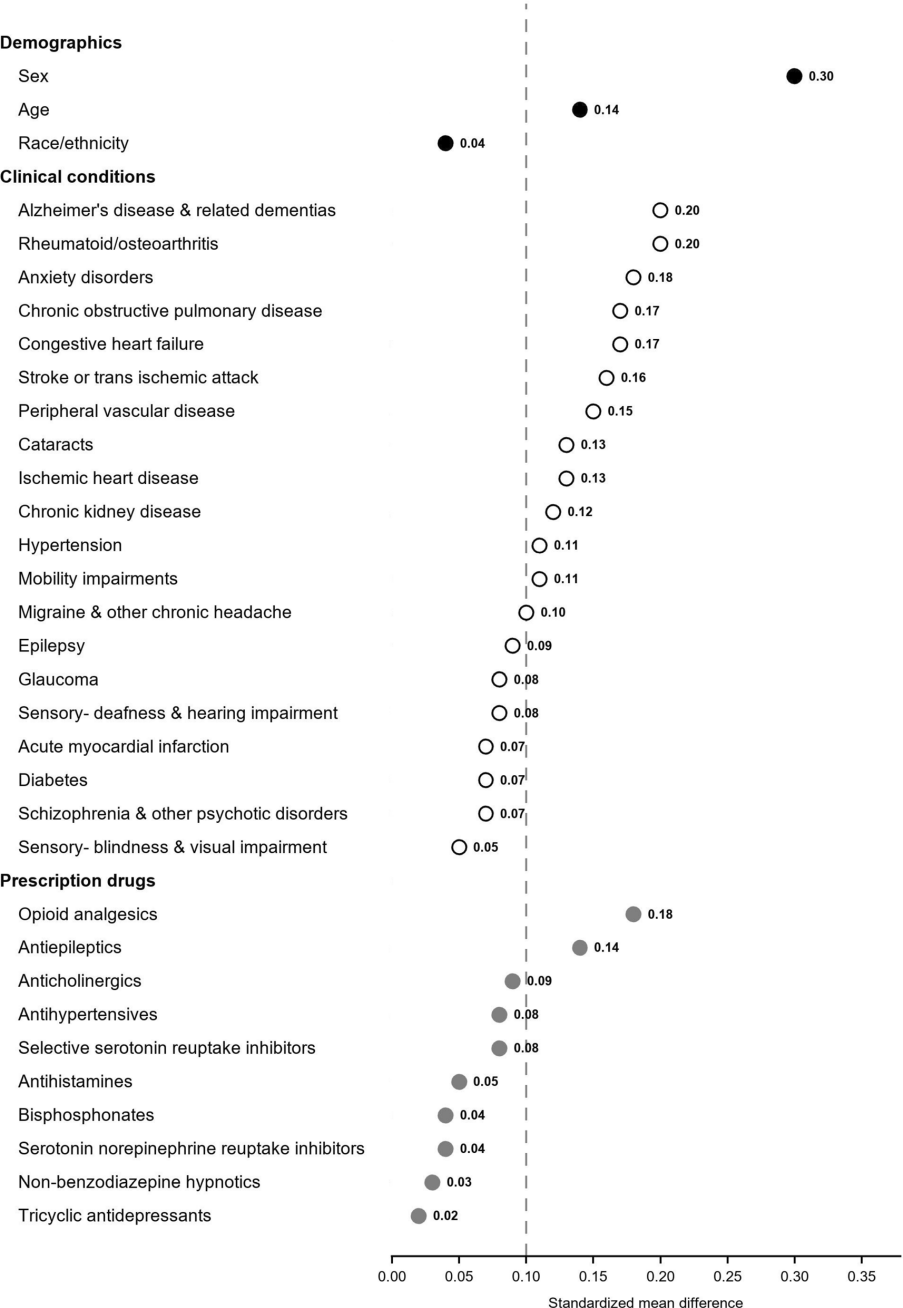
Absolute value of standardized mean difference between police crash reports and Medicare claims by demographics, comorbidities, and medications, 2008–2017

*Characteristics of crash-involved drivers by data source:* When Medicare claims included all eligible claims irrespective of the injured party’s designated role in the crash, compared to all crash-involved drivers with a police report, those with a Medicare claim were more likely to be female (62.4% vs 51.8%, standardized mean difference [SMD] = 0.30), slightly older (77.2 vs. 76.5, SMD = 0.14) and more likely to have a number of clinical diagnoses prior to the crash, including Alzheimer’s disease and related dementias (13.0% vs 9.2% SMD = 0.20), rheumatoid arthritis/osteoarthritis (69.5% vs 61.4%, SMD = 0.20), anxiety (22.9% vs 17.0%, SMD = 0.18), chronic obstructive pulmonary disease (31.6% vs. 25.5%, SMD = 0.17), congestive heart failure (35.6% vs 29.9%, SMD = 0.17), stroke or transient ischemic attack (18.6% vs. 14.5%, SMD = 0.16), peripheral vascular disease (35.6% vs. 30.2%, SMD = 0.15), cataracts (78.1% vs. 74.2%, SMD = 0.13), ischemic heart disease (66.7% vs. 61.6%, SMD = 0.13), chronic kidney disease (28.1% vs. 24.3%, SMD = 0.12), hypertension (90.1% vs. 86.6%, SMD = 0.11), and mobility impairments (3.3% vs 2.2%, SMD = 0.11). Those with a Medicare claim for a crash-related injury were also more likely to have a dispensing for an opioid (33.8% vs 27.6%, SMD = 0.18) or an antiepileptic (12.9% vs 9.6%, SMD = 0.14) prior to the crash (Table 2 and Fig. 3).

**Fig. 4:**
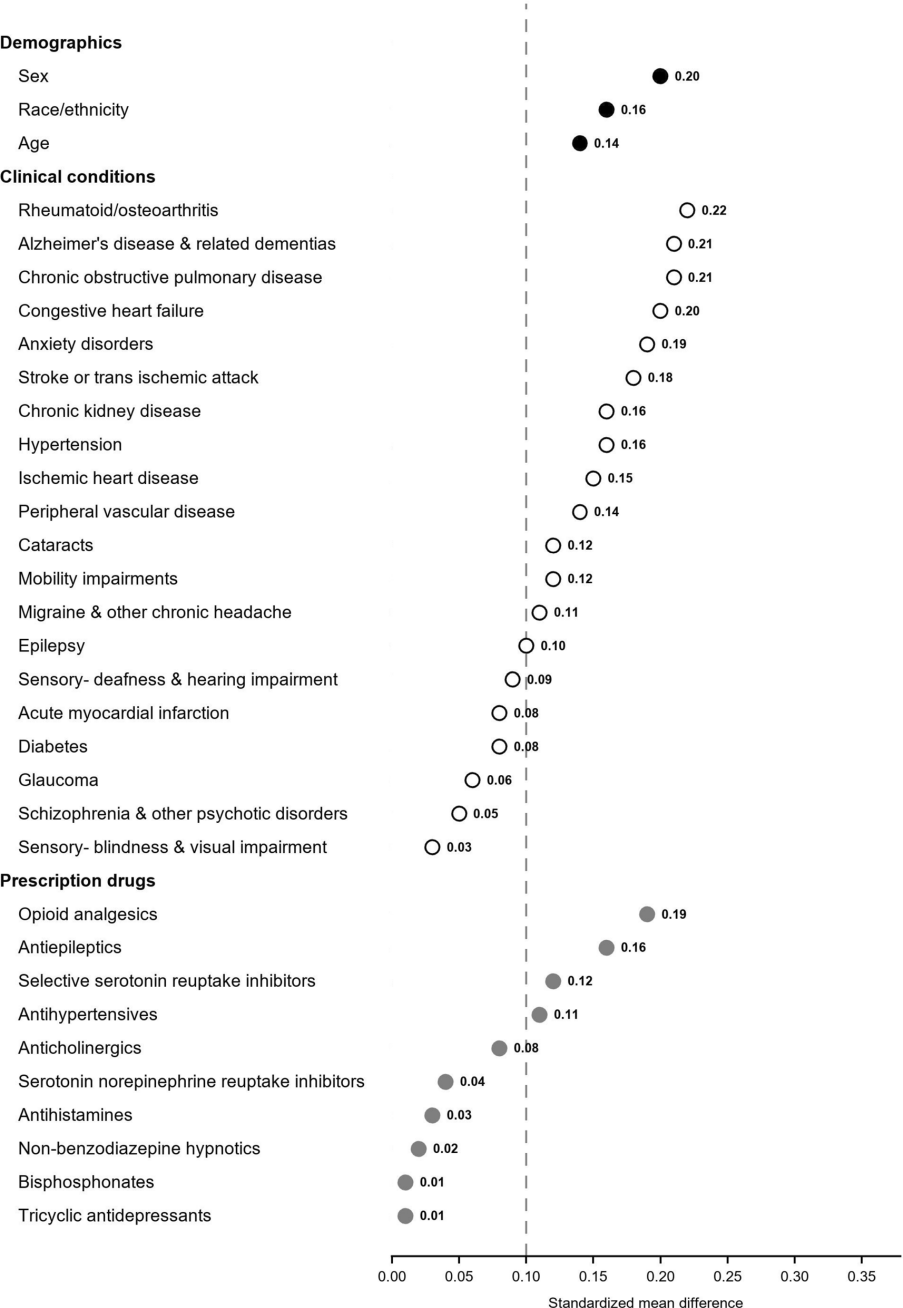
Absolute value of standardized mean difference between police crash reports and Medicare claims in which the claim specifies the injured party was the driver or left unspecified by demographics, comorbidities, and medications, 2008–2017

Our findings were similar when Medicare claims were limited to claims in which the injured party was designated the driver or left unspecified, with the exception that crash-involved drivers with a Medicare claim were more likely than those with a police report to be Black or African American (8.9% vs. 6.8%, SMD = 0.16) and were more likely to have a dispensing for a selective serotonin reuptake inhibitor (SSRI; 15.9% vs. 13.0%, SMD = 0.12) or an antihypertensive (78.9% vs 75.7%, SMD = 0.11) in the year prior to the crash (Table 3 and Fig. 4).

**Table 3:** Characteristics of crash-involved drivers by source(s) used to identify the crash (driver or unspecified only)

	Police-reported crash	Medicare claim for a crash-related injury
N (% of all crash-involved drivers)^a^	116,342 (97.6%)	9,679 (8.1%)
*Demographics*		
Age, years, mean (SD)	76.5 (6.3)	77.2 (6.5)
*Sex*		
Male	56,032 (48.2%)	3,864 (39.9%)
Female	60,310 (51.8%)	5,815 (60.1%)
*Race/ethnicity*		
Black/African American	7,926 (6.8%)	857 (8.9%)
White	101,088 (86.9%)	8,342 (86.2%)
Asian	2,498 (2.1%)	180 (1.9%)
Hispanic	1,483 (1.3%)	115 (1.2%)
Unknown/Other	3,347 (2.9%)	185 (1.9%)
*Clinical conditions diagnosed prior to the crash*		
Acute myocardial infarction	5,404 (4.6%)	546 (5.6%)
Alzheimer’s disease and related dementias	10,663 (9.2%)	1,215 (12.6%)
Anxiety disorders	19,737 (17.0%)	2,208 (22.8%)
Cataracts	86,279 (74.2%)	7,518 (77.7%)
Chronic kidney disease	28,210 (24.2%)	2,747 (28.4%)
Chronic obstructive pulmonary disease	29,642 (25.5%)	3,069 (31.7%)
Congestive heart failure	34,798 (29.9%)	3,428 (35.4%)
Diabetes	55,614 (47.8%)	4,994 (51.6%)
Epilepsy	2,074 (1.8%)	260 (2.7%)
Glaucoma	37,250 (32.0%)	3,293 (34.0%)
Hypertension	100,993 (86.8%)	8,755 (90.5%)
Ischemic heart disease	71,613 (61.6%)	6,475 (66.9%)
Migraine and other chronic headache	4,348 (3.7%)	501 (5.2%)
Mobility impairments	2,603 (2.2%)	302 (3.1%)
Peripheral vascular disease	35,120 (30.2%)	3,385 (35.0%)
Rheumatoid arthritis/Osteoarthritis	71,443 (61.4%)	6,722 (69.4%)
Schizophrenia and other psychotic disorders	1,574 (1.4%)	175 (1.8%)
Blindness and visual impairment	245 (0.2%)	23 (0.2%)
Deafness and hearing impairment	19,488 (16.8%)	1,784 (18.4%)
Stroke or transient ischemic attack	16,902 (14.5%)	1,765 (18.2%)
*Prescription drug dispensing in the 12 months prior to the crash*		
Selective serotonin reuptake inhibitors	15,151 (13.0%)	1,541 (15.9%)
Serotonin norepinephrine reuptake inhibitors	4,196 (3.6%)	418(4.3%)
Tricyclic antidepressants	2,582 (2.2%)	252 (2.6%)
Antiepileptics	11,180 (9.6%)	1,256 (13.0%)
Anticholinergics	21,140 (18.2%)	2,045 (21.1%)
Antihypertensives	88,091 (75.7%)	7,638 (78.9%)
Antihistamines	10,367 (8.9%)	943 (9.7%)
Bisphosphonates	7,835 (6.7%)	699 (7.2%)
Opioid analgesics	32,051 (27.5%)	3,249 (33.6%)
Non-benzodiazepine hypnotics	8,974 (7.7%)	820 (8.5%)

^a^Percentages add up to more than 100 because some crash-involved drivers have both a police report and a Medicare claim for a crash-related injury

SD = standard deviation

Note that claims are limited to those in which the injured party is identified as the driver or left unspecified

## Discussion

Using 10 years of Medicare insurance claims linked to police crash reports in the state of New Jersey, we found substantial differences in the characteristics of crash-involved drivers depending on the source used to identify them. Importantly, we also found that our results were largely the same even when limiting Medicare claims to those specifically designating the injured individual as the driver–an uncommon practice in studies that use administrative healthcare data.

Although our goal was not to identify specific causes of crash or crash-related injury, our findings have important implications for investigators attempting to do so. First, it is possible that the differences in the characteristics of crash-involved drivers with and without an injury do in fact reflect different causal mechanisms. For example, the greater percentage of injured drivers with an opioid dispensing in the past year could indicate that opioid use has a direct effect on injury that is not (fully) mediated through its effect on the risk of a crash. Second, a key step in answering any causal question using observational data is to identify the variables required to adjust for confounding. When an investigator intends to estimate the causal effect of a driver-level characteristic on crash, they base the choice of confounders on their substantive understanding of the relationship between the exposure and a crash. Administrative healthcare data, however, only contain information on crash-related injuries. If the confounders of the exposure-crash and exposure-crash-related-injury relationships differ (as our results suggest may be the case), then by conflating crash and crash-related injury investigators risk conditioning on a wrong or incomplete set of variables. The result is that their estimate neither has the interpretation the investigator intended (the causal effect on crash) nor is it an unbiased estimate of a different causal effect that they did not intend to estimate (the causal effect on crash-related injury).

Even when the goal is not causal, the distinction between crash and crash-related injury as an outcome is important for developing effective policy. For instance, several states impose license restrictions on “medically-at-risk drivers,” such as where and under what circumstances they can drive (e.g., how far from home, only during certain hours, not on highways). (Graham and Darrah 2020) Though some of these policies are effective at reducing crash-related injury by reducing the risk of a crash, (Lococo et al. 2013; Braitman et al. 2010) they can also severely limit mobility, putting older adults at risk of social isolation and depression. (Chihuri et al. 2016; Curl et al. 2014; Qin et al. 2019) In contrast, if a particular condition is not associated with an increased risk of a crash, but instead places the driver at increased risk of an injury once involved in a crash, then interventions to reduce the risk of injury, such as enhanced seat belt technology, may have a greater impact on crash-related injury without unnecessarily affecting mobility.

Our study has limitations worth mentioning. First, while our findings suggest that the characteristics of crash-involved drivers with and without a Medicare claim for a crash-related injury differ overall, we did not test a hypothesis about any particular exposure. Thus, while we can speculate about the potential for bias, the presence and extent of this bias will vary across different exposures. Second, our police-reported crashes are limited to New Jersey, which may not be representative of other states. More importantly, however, we are unable to identify police-reported crashes that occur outside of New Jersey. This could be an issue if licensed drivers with a home residence in New Jersey spend significant amounts of time in other states, in which case, we may be missing a significant number of crashes and crash-related injuries concentrated in a possibly highly selected group of individuals (e.g., those who are well enough and have the means to travel and spend time somewhere other than their home state). Although we cannot identify these out-of-state crashes, our approach reflects the way analyses occur in applied research based in the United States, where there is no national database of police-reported crashes and analyses are most often limited to one state. Last, our analysis of the NJ-SHO-Medicare linkage was limited to Medicare fee-for-service beneficiaries and did not include anyone enrolled in Medicare Advantage, which makes up 37% of the Medicare population in New Jersey. (Ochieng et al. 2023) However, our findings are meant to highlight the importance of distinguishing crash from crash-related injury in an analysis. Thus, while the actual demographic and clinical differences between crash and crash-related drivers might differ in the Medicare Advantage population (or any other non-overlapping population), we have no reason to believe that we would no longer observe differences between crash-involved drivers identified from police reports and those identified from Medicare claims.

## Conclusion

Ultimately, the choice between crash and crash-related injury as an outcome reflects two fundamentally different research questions with different analytic and policy implications. Our findings show that—despite a growing trend in traffic safety research using administrative healthcare data to conflate the two—crash and crash-related injury are not interchangeable outcomes. To identify the most effective mechanisms for reducing morbidity and mortality from crashes, especially among older drivers, researchers should consider augmenting administrative datasets with information from police crash reports, and vice versa. When those data are not available, we caution researchers and policymakers against the tendency to conflate crash and crash-related injury when interpreting their findings.

### Supplementary Information

Supplementary Material 1.

## Data Availability

The linked data are not available due to privacy restrictions. Investigators interested in obtaining the data may apply directly to the Centers for Medicare and Medicaid Services and the New Jersey Motor Vehicle Commission. The code can be made available to researchers upon request by emailing the corresponding author. In compliance with our funder’s Resource Sharing Policy, while person level data cannot be shared due to DUA restrictions, additional information about the data and methods used for these analyses can be found in Brown’s Digital Repository (doi: 10.26300/985b-8j02).

## Abbreviations

CCW: Chronic conditions warehouse
NJ-SHO: New Jersey safety and health outcomes
MBSF: Medicare master beneficiary summary file
TBI: Traumatic brain injury
SD: Standard deviation
