# Supplementary material for: Implications of using administrative healthcare data to identify risk of motor vehicle crash-related injury: the importance of distinguishing crash from crash-related injury
Source: Inj Epidemiol. 2024 Aug 12;11:38. doi: 10.1186/s40621-024-00523-3 (PMC11318118; doi:10.1186/s40621-024-00523-3)
Supplement: Supplementary file 1 — Supplementary Material 1. [file 40621_2024_523_MOESM1_ESM.docx]

**Supplementary Material**

| **Supplementary table 1.** Characteristics of all crash-involved drivers by the source(s) used to identify the crash across mutually exclusive sources for identifying the crash-involved driver, where all claims are included irrespective of the injured party’s designated role in the crash 2008 -2017 (N = 121,867). | | | | | |
| --- | --- | --- | --- | --- | --- |
|  | **Police-reported crash only** | **Medicare claim for a crash-related injury only** | **Both a police-report and a Medicare claim for a crash-related injury** | **p-value** | |
| **N (% of all crash-involved individuals)** | 109,566 (89.9%) | 5,402 (4.4%) | 6,899 (5.7%) |  | |
| **Demographics** |  |  |  |  | |
| Age (mean/SD) | 76.5 (6.3) | 77.3 (6.6) | 77.1 (6.5) | <.0001 | |
| Sex |  |  |  | <.0001 | |
| Male | 53,292 (48.6%) | 1,834 (34.0%) | 2,796 (40.5%) |  |  |
| Female | 56,274 (51.4%) | 3,568 (66.0%) | 4,103 (59.5%) |  |  |
| Race/ethnicity |  |  |  | <.0001 | |
| Black/African American | 7,332 (6.7%) | 449 (8.3%) | 615 (8.9%) |  |  |
| White | 95,250 (86.9%) | 4,643 (85.9%) | 5,932 (86.0%) |  |  |
| Asian | 2,366 (2.2%) | 103 (1.9%) | 136 (2.0%) |  |  |
| Hispanic | 1,400 (1.3%) | 76 (1.4%) | 83 (1.2%) |  |  |
| Unknown/Other | 3,218 (2.9%) | 131 (2.4%) | 133 (1.9%) |  |  |
| **Clinical conditions diagnosed prior to the crash** |  |  |  |  | |
| Acute myocardial infarction | 5,046 (4.6%) | 330 (6.1%) | 362 (5.2%) | <.0001 | |
| Alzheimer’s disease and related dementias | 9,907 (9.0%) | 833 (15.4%) | 772 (11.2%) | <.0001 | |
| Anxiety disorders | 18,238 (16.6%) | 1,285 (23.8%) | 1,528 (22.1%) | <.0001 | |
| Cataracts | 81,051 (74.0%) | 4,292 (79.5%) | 5,311 (77.0%) | <.0001 | |
| Chronic kidney disease | 26,364 (24.1%) | 1,579 (29.2%) | 1,880 (27.3%) | <.0001 | |
| Chronic obstructive pulmonary disease | 27,576 (25.2%) | 1,772 (32.8%) | 2,110 (30.6%) | <.0001 | |
| Congestive heart failure | 32,509 (29.7%) | 2,048 (37.9%) | 2,331 (33.8%) | <.0001 | |
| Diabetes | 52,121 (47.6%) | 2,763 (51.1%) | 3,558 (51.6%) | <.0001 | |
| Epilepsy | 1,909 (1.7%) | 171 (3.2%) | 168 (2.4%) | <.0001 | |
| Glaucoma | 34,968 (31.9%) | 1,920 (35.5%) | 2,326 (33.7%) | <.0001 | |
| Hypertension | 94,893 (86.6%) | 4,872 (90.2%) | 6,213 (90.1%) | <.0001 | |
| Ischemic heart disease | 67,129 (61.3%) | 3,635 (67.3%) | 4,569 (66.2%) | <.0001 | |
| Migraine and other chronic headache | 4,022 (3.7%) | 313 (5.8%) | 330 (4.8%) | <.0001 | |
| Mobility impairments | 2,427 (2.2%) | 223 (4.1%) | 181 (2.6%) | <.0001 | |
| Peripheral vascular disease | 32,800 (29.9%) | 2,010 (37.2%) | 2,371 (34.4%) | <.0001 | |
| Rheumatoid/Osteoarthritis | 66,797 (61.0%) | 3,812 (70.6%) | 4,734 (68.6%) | <.0001 | |
| Schizophrenia and other psychotic disorders | 1,457 (1.3%) | 120 (2.2%) | 118 (1.7%) | <.0001 | |
| Sensory - blindness and visual impairment | 233 (0.2%) | 28 (0.5%) | 12 (0.2%) | <.0001 | |
| Sensory - deafness and hearing impairment | 18,287 (16.7%) | 1,073 (19.9%) | 1,229 (17.8%) | <.0001 | |
| Stroke or trans ischemic attack | 15,753 (14.4%) | 1,106 (20.5%) | 1,176 (17.0%) | <.0001 | |
| **Prescription drug dispensing in the 12-months prior to the crash** |  |  |  |  | |
| Selective serotonin reuptake inhibitors | 14,105 (12.9%) | 854 (15.8%) | 1,059 (15.4%) | <.0001 | |
| Serotonin norepinephrine reuptake inhibitors | 3,904 (3.6%) | 235 (4.4%) | 299 (4.3%) | <.0001 | |
| Tricyclic antidepressants | 2,398 (2.2%) | 133 (2.5%) | 187 (2.7%) | 0.0087 | |
| Antiepileptics | 10,353 (9.4%) | 744 (13.8%) | 840 (12.2%) | <.0001 | |
| Anticholinergics | 19,713 (18.0%) | 1,166 (21.6%) | 1,457 (21.1%) | <.0001 | |
| Antihypertensives | 82,774 (75.5%) | 4,255 (78.8%) | 5,419 (78.5%) | <.0001 | |
| Antihistamines | 9,707 (8.9%) | 560 (10.4%) | 675 (9.8%) | <.0001 | |
| Bisphosphonates | 7,326 (6.7%) | 419 (7.8%) | 514 (7.5%) | 0.0007 | |
| Opioid analgesics | 29,843 (27.2%) | 1,909 (35.3%) | 2,252 (32.6%) | <.0001 | |
| Non-benzodiazepine hypnotics | 8,396 (7.7%) | 464 (8.6%) | 592 (8.6%) | 0.0014 | |
| ^a^ We used a chi-square test to compare proportions across groups with the exception of mean age for which we used an Analysis of Variance (ANOVA) to compare means.  SD = standard deviation | | | | |  |

| **Supplementary table 2.** Characteristics of all crash-involved drivers by the source(s) used to identify the crash across mutually exclusive sources for identifying the crash-involved driver, where claims are limited to those in which the injured party is identified as the driver or left unspecified 2008 -2017 (N = 119,245). | | | | | | |
| --- | --- | --- | --- | --- | --- | --- |
|  | **Police-reported crash only** | **Medicare claim for a crash-related injury only** | **Both a police-report and a Medicare claim for a crash-related injury** | **p-value** | |  |
| **N (% of all crash-involved individuals)** | 109,566 (91.9%) | 2,903 (2.4%) | 6,776 (5.7%) |  | |  |
| **Demographics** |  |  |  |  | |  |
| Age (mean/SD) | 76.5 (6.3) | 77.4 (6.7) | 77.1 (6.5) | <.0001 | |  |
| Sex |  |  |  | <.0001 | |  |
| Male | 53,292 (48.6%) | 1124 (38.7%) | 2,740 (40.4%) |  |  |  |
| Female | 56,274 (51.4%) | 1779 (61.3%) | 4,036 (59.6%) |  |  |  |
| Race/ethnicity |  |  |  | <.0001 | |  |
| Black/African American | 7,332 (6.7%) | 263 (9.1%) | 594 (8.8%) |  |  |  |
| White | 95,250 (86.9%) | 2,504 (86.3%) | 5,838 (86.2%) |  |  |  |
| Asian | 2,366 (2.2%) | 48 (1.7%) | 132 (1.9%) |  |  |  |
| Hispanic | 1,400 (1.3%) | 32 (1.1%) | 83 (1.2%) |  |  |  |
| Unknown/Other | 3,218 (2.9%) | 56 (1.9%) | 129 (1.9%) |  |  |  |
| **Clinical conditions diagnosed prior to the crash** |  |  |  |  | |  |
| Acute myocardial infarction | 5,046 (4.6%) | 188 (6.5%) | 358 (5.3%) | <.0001 | |  |
| Alzheimer’s disease and related dementias | 9,907 (9.0%) | 459 (15.8%) | 756 (11.2%) | <.0001 | |  |
| Anxiety disorders | 18,238 (16.6%) | 709 (24.4%) | 1,499 (22.1%) | <.0001 | |  |
| Cataracts | 81,051 (74.0%) | 2,290 (78.9%) | 5,228 (77.2%) | <.0001 | |  |
| Chronic kidney disease | 26,364 (24.1%) | 901 (31.0%) | 1,846 (27.2%) | <.0001 | |  |
| Chronic obstructive pulmonary disease | 27,576 (25.2%) | 1,003 (34.6%) | 2,066 (30.5%) | <.0001 | |  |
| Congestive heart failure | 32,509 (29.7%) | 1,139 (39.2%) | 2,289 (33.8%) | <.0001 | |  |
| Diabetes | 52,121 (47.6%) | 1,501 (51.7%) | 3,493 (51.5%) | <.0001 | |  |
| Epilepsy | 1,909 (1.7%) | 95 (3.3%) | 165 (2.4%) | <.0001 | |  |
| Glaucoma | 34,968 (31.9%) | 1,011 (34.8%) | 2,282 (33.7%) | 0.0002 | |  |
| Hypertension | 94,893 (86.6%) | 2,655 (91.5%) | 6,100 (90.0%) | <.0001 | |  |
| Ischemic heart disease | 67,129 (61.3%) | 1,991 (68.6%) | 4,484 (66.2%) | <.0001 | |  |
| Migraine and other chronic headache | 4,022 (3.7%) | 175 (6.0%) | 326 (4.8%) | <.0001 | |  |
| Mobility impairments | 2,427 (2.2%) | 126 (4.3%) | 176 (2.6%) | <.0001 | |  |
| Peripheral vascular disease | 32,800 (29.9%) | 1,065 (36.7%) | 2,320 (34.2%) | <.0001 | |  |
| Rheumatoid/Osteoarthritis | 66,797 (61.0%) | 2,076 (71.5%) | 4,646 (68.6%) | <.0001 | |  |
| Schizophrenia and other psychotic disorders | 1,457 (1.3%) | 58 (2.0%) | 117 (1.7%) | 0.0003 | |  |
| Sensory - blindness and visual impairment | 233 (0.2%) | 11 (0.4%) | 12 (0.2%) | 0.1273 | |  |
| Sensory - deafness and hearing impairment | 18,287 (16.7%) | 583 (20.1%) | 1,201 (17.7%) | <.0001 | |  |
| Stroke or trans ischemic attack | 15,753 (14.4%) | 616 (21.2%) | 1,149 (17.0%) | <.0001 | |  |
| **Prescription drug dispensing in the 12-months prior to the crash** |  |  |  |  | |  |
| Selective serotonin reuptake inhibitors | 14,105 (12.9%) | 495 (17.1%) | 1,046 (15.4%) | <.0001 | |  |
| Serotonin norepinephrine reuptake inhibitors | 3,904 (3.6%) | 126 (4.3%) | 292 (4.3%) | 0.0007 | |  |
| Tricyclic antidepressants | 2,398 (2.2%) | 68 (2.3%) | 184 (2.7%) | 0.0154 | |  |
| Antiepileptics | 10,353 (9.4%) | 429 (14.8%) | 827 (12.2%) | <.0001 | |  |
| Anticholinergics | 19,713 (18.0%) | 618 (21.3%) | 1,427 (21.1%) | <.0001 | |  |
| Antihypertensives | 82,774 (75.5%) | 2,321 (80.0%) | 5,317 (78.5%) | <.0001 | |  |
| Antihistamines | 9,707 (8.9%) | 283 (9.7%) | 660 (9.7%) | 0.0141 | |  |
| Bisphosphonates | 7,326 (6.7%) | 190 (6.5%) | 509 (7.5%) | 0.0289 | |  |
| Opioid analgesics | 29,843 (27.2%) | 1,041 (35.9%) | 2,208 (32.6%) | <.0001 | |  |
| Non-benzodiazepine hypnotics | 8,396 (7.7%) | 242 (8.3%) | 578 (8.5%) | 0.0160 | |  |
| ^a^ We used a chi-square test to compare proportions across groups with the exception of mean age for which we used an Analysis of Variance (ANOVA) to compare means.  SD = standard deviation | | | | |  |  |

**Table 3**

| **Supplementary table 3.** ICD-9 and ICD-10 codes used to define clinical conditions in the chronic conditions warehouse. | | |
| --- | --- | --- |
| **Clinical condition** | **ICD-9 codes** | **ICD-10 codes** |
| Acute myocardial infarction | 410.01, 410.11, 410.21, 410.31, 410.41, 410.51, 410.61, 410.71, 410.81, 410.91 (ONLY first or second DX on the claim) | I21.01, I21.02, I21.09, I21.11, I21.19, I21.21, I21.29, I21.3, I21.4, I21.9, I21.A1, I21.A9, I22.0, I22.1, I22.2, I22.8, I22.9, I23.0, I23.1, I23.2, I23.3, I23.4, I23.5, I23.6, I23.7, I23.8 (any DX on the claim) |
| Alzheimer’s disease and related dementias | 331.0, 331.11, 331.19, 331.2, 331.7, 290.0, 290.10, 290.11, 290.12, 290.13, 290.20, 290.21, 290.3, 290.40, 290.41, 290.42, 290.43, 294.0, 294.10, 294.11, 294.20, 294.21, 294.8, 797 (any DX on the claim) | G30.0, G30.1, G30.8, G30.9 (any DX on the claim) |
| Anxiety disorders | DX 293.84, 300.00, 300.01, 300.02, 300.09, 300.10, 300.20, 300.21, 300.22, 300.23, 300.29, 300.3, 300.5, 300.89, 300.9, 308.0, 308.1, 308.2, 308.3, 308.4, 308.9, 309.81, 313.0, 313.1, 313.21, 313.22, 313.3, 313.82, 313.83 (any DX on the claim | DX F06.4, F40.00, F40.01, F40.02, F40.10, F40.11, F40.210, F40.218, F40.220, F40.228, F40.230, F40.231, F40.232, F40.233, F40.240, F40.241, F40.242, F40.243, F40.248, F40.290, F40.291, F40.298, F40.8, F40.9, F41.0, F41.1, F41.3, F41.8, F41.9, F42, F42.2, F42.3, F42.4, F42.8, F42.9, F43.0, F43.10, F43.11, F43.12, F44.9, F45.8, F48.8, F48.9, F93.8, F99, R45.2, R45.5, R45.6, R45.7 (any DX on the claim) |
| Cataracts | 366.01, 366.02, 366.03, 366.04, 366.09, 366.10, 366.12, 366.13, 366.14, 366.15, 366.16, 366.17, 366.18, 366.19, 366.20, 366.21, 366.22, 366.23, 366.30, 366.45, 366.46, 366.50, 366.51, 366.52, 366.53, 366.8, 366.9, 379.26, 379.31, 379.39, 743.30, 743.31, 743.32, 743.33, V43.1 (ONLY principal DX on the claim) | E08.36, E09.36, E10.36, E11.36, E13.36, H25.011, H25.012, H25.013, H25.019, H25.031, H25.032, H25.033, H25.039, H25.041, H25.042, H25.043, H25.049, H25.091, H25.092, H25.093, H25.099, H25.10, H25.11, H25.12, H25.13, H25.20, H25.21, H25.22, H25.23, H25.811, H25.812, H25.813, H25.819, H25.89, H25.9, H26.001, H26.002, H26.003, H26.009, H26.011, H26.012, H26.013, H26.019, H26.031, H26.032, H26.033, H26.039, H26.041, H26.042, H26.043, H26.049, H26.051, H26.052, H26.053, H26.059, H26.061, H26.062, H26.063, H26.069, H26.09, H26.101, H26.102, H26.103, H26.109, H26.111, H26.112, H26.113, H26.119, H26.121, H26.122, H26.123, H26.129, H26.131, H26.132, H26.133, H26.139, H26.20, H26.211, H26.212, H26.213, H26.219, H26.221, H26.222, H26.223, H26.229, H26.30, H26.31, H26.32, H26.33, H26.40, H26.411, H26.412, H26.413, H26.419, H26.491, H26.492, H26.493, H26.499, H26.8, H26.9, Q12.0 (any DX on the claim) |
| Chronic kidney disease | 016.00, 016.01, 016.02, 016.03, 016.04, 016.05, 016.06, 095.4, 189.0, 189.9, 223.0, 236.91, 249.40, 249.41, 250.40, 250.41, 250.42, 250.43, 271.4, 274.10, 283.11, 403.01, 403.11, 403.91, 404.02, 404.03, 404.12, 404.13, 404.92, 404.93, 440.1, 442.1, 572.4, 580.0, 580.4, 580.81, 580.89, 580.9, 581.0, 581.1, 581.2, 581.3, 581.81, 581.89, 581.9, 582.0, 582.1, 582.2, 582.4, 582.81, 582.89, 582.9, 583.0, 583.1, 583.2, 583.4, 583.6, 583.7, 583.81, 583.89, 583.9, 584.5, 584.6, 584.7, 584.8, 584.9, 585.1, 585.2, 585.3, 585.4, 585.5, 585.6, 585.9, 586, 587, 588.0, 588.1, 588.81, 588.89, 588.9, 591, 753.12, 753.13, 753.14, 753.15, 753.16, 753.17, 753.19, 753.20, 753.21, 753.22, 753.23, 753.29, 794.4 (any DX on the claim) | A18.11, A52.75, B52.0, E08.21, E08.22, E08.29, E09.21, E09.22, E09.29, E10.21, E10.22, E10.29, E11.21, E11.22, E11.29, E13.21, E13.22, E13.29, I12.0, I12.9, I13.0, I13.10, I13.11, I13.2, K76.7, M10.30, M10.311, M10.312, M10.319, M10.321, M10.322, M10.329, M10.331, M10.332, M10.339, M10.341, M10.342, M10.349, M10.351, M10.352, M10.359, M10.361, M10.362, M10.369, M10.371, M10.372, M10.379, M10.38, M10.39, M32.14, M32.15, M35.04, M35.0A, N01.0, N01.1, N01.2, N01.3, N01.4, N01.5, N01.6, N01.7, N01.8, N01.9, N01.A, N02.0, N02.1, N02.2, N02.3, N02.4, N02.5, N02.6, N02.7, N02.8, N02.9, N02.A, N03.0, N03.1, N03.2, N03.3, N03.4, N03.5, N03.6, N03.7, N03.8, N03.9, N03.A, N04.0, N04.1, N04.2, N04.3, N04.4, N04.5, N04.6, N04.7, N04.8, N04.9, N04.A, N05.0, N05.1, N05.2, N05.3, N05.4, N05.5, N05.6, N05.7, N05.8, N05.9, N05.A, N06.0, N06.1, N06.2, N06.3, N06.4, N06.5, N06.6, N06.7, N06.8, N06.9, N06.A, N07.0, N07.1, N07.2, N07.3, N07.4, N07.5, N07.6, N07.7, N07.8, N07.9, N07.A, N08, N14.0, N14.1, N14.11, N14.19, N14.2, N14.3, N14.4, N15.0, N15.8, N15.9, N16, N18.1, N18.2, N18.3, N18.30, N18.31, N18.32, N18.4, N18.5, N18.6, N18.9, N25.1, N25.89, N25.9, N26.1, N26.9, N99.0, Q61.02, Q61.11, Q61.19, Q61.2, Q61.3, Q61.4, Q61.5, Q61.8 (any DX on the claim) |
| Chronic obstructive pulmonary disease | 490, 491.0, 491.1, 491.20, 491.21, 491.22, 491.8, 491.9, 492.0, 492.8, 494.0, 494.1, 496 (any DX on the claim) | J40, J41.0, J41.1, J41.8, J42, J43.0, J43.1, J43.2, J43.9, J44.0, J44.1, J44.9, J47.0, J47.1, J47.9, J98.2, J98.3 (any DX on the claim) |
| Congestive heart failure | 398.91, 402.01, 402.11, 402.91, 404.01, 404.03, 404.11, 404.13, 404.91, 404.93, 428.0, 428.1, 428.20, 428.21, 428.22, 428.23, 428.30, 428.31, 428.32, 428.33, 428.40, 428.41, 428.42, 428.43, 428.9 (any DX on the claim) | I09.81, I11.0, I13.0, I13.2, I42.0, I42.5, I42.6, I42.7, I42.8, I43, I50.1, I50.20, I50.21, I50.22, I50.23, I50.30, I50.31, I50.32, I50.33, I50.40, I50.41, I50.42, I50.43, I50.810, I50.811, I50.812, I50.813, I50.814, I50.82, I50.83, I50.84, I50.89, I50.9, P29.0 (any DX on the claim) |
| Diabetes | 249.00, 249.01, 249.10, 249.11, 249.20, 249.21, 249.30, 249.31, 249.40, 249.41, 249.50, 249.51, 249.60, 249.61, 249.70, 249.71, 249.80, 249.81, 249.90, 249.91, 250.00, 250.01, 250.02, 250.03, 250.10, 250.11, 250.12, 250.13, 250.20, 250.21, 250.22, 250.23, 250.30, 250.31, 250.32, 250.33, 250.40, 250.41, 250.42, 250.43, 250.50, 250.51, 250.52, 250.53, 250.60, 250.61, 250.62, 250.63, 250.70, 250.71, 250.72, 250.73, 250.80, 250.81, 250.82, 250.83, 250.90, 250.91, 250.92, 250.93, 357.2, 362.01, 362.02, 362.03, 362.04, 362.05, 362.06, 366.41 (any DX on the claim) | E08.00, E08.01, E08.10, E08.11, E08.21, E08.22, E08.29, E08.311, E08.319, E08.321, E08.3211, E08.3212, E08.3213, E08.3219, E08.329, E08.3291, E08.3292, E08.3293, E08.3299, E08.331, E08.3311, E08.3312, E08.3313, E08.3319, E08.339, E08.3391, E08.3392, E08.3393, E08.3399, E08.341, E08.3411, E08.3412, E08.3413, E08.3419, E08.349, E08.3491, E08.3492, E08.3493, E08.3499, E08.351, E08.3511, E08.3512, E08.3513, E08.3519, E08.3521, E08.3522, E08.3523, E08.3529, E08.3531, E08.3532, E08.3533, E08.3539, E08.3541, E08.3542, E08.3543, E08.3549, E08.3551, E08.3552, E08.3553, E08.3559, E08.359, E08.3591, E08.3592, E08.3593, E08.3599, E08.36, E08.37X1, E08.37X2, E08.37X3, E08.37X9, E08.39, E08.40, E08.41, E08.42, E08.43, E08.44, E08.49, E08.51, E08.52, E08.59, E08.610, E08.618, E08.620, E08.621, E08.622, E08.628, E08.630, E08.638, E08.641, E08.649, E08.65, E08.69, E08.8, E08.9, E09.00, E09.01, E09.10, E09.11, E09.21, E09.22, E09.29, E09.311, E09.319, E09.321, E09.3211, E09.3212, E09.3213, E09.3219, E09.329, E09.3291, E09.3292, E09.3293, E09.3299, E09.331, E09.3311, E09.3312, E09.3313, E09.3319, E09.339, E09.3391, E09.3392, E09.3393, E09.3399, E09.341, E09.3411, E09.3412, E09.3413, E09.3419, E09.349, E09.3491, E09.3492, E09.3493, E09.3499, E09.351, E09.3511, E09.3512, E09.3513, E09.3519, E09.3521, E09.3522, E09.3523, E09.3529, E09.3531, E09.3532, E09.3533, E09.3539, E09.3541, E09.3542, E09.3543, E09.3549, E09.3551, E09.3552, E09.3553, E09.3559, E09.359, E09.3591, E09.3592, E09.3593, E09.3599, E09.36, E09.37X1, E09.37X2, E09.37X3, E09.37X9, E09.39, E09.40, E09.41, E09.42, E09.43, E09.44, E09.49, E09.51, E09.52, E09.59, E09.610, E09.618, E09.620, E09.621, E09.622, E09.628, E09.630, E09.638, E09.641, E09.649, E09.65, E09.69, E09.8, E09.9, E10.10, E10.11, E10.21, E10.22, E10.29, E10.311, E10.319, E10.321, E10.3211, E10.3212, E10.3213, E10.3219, E10.329, E10.3291, E10.3292, E10.3293, E10.3299, E10.331, E10.3311, E10.3312, E10.3313, E10.3319, E10.339, E10.3391, E10.3392, E10.3393, E10.3399, E10.341, E10.3411, E10.3412, E10.3413, E10.3419, E10.349, E10.3491, E10.3492, E10.3493, E10.3499, E10.351, E10.3511, E10.3512, E10.3513, E10.3519, E10.3521, E10.3522, E10.3523, E10.3529, E10.3531, E10.3532, E10.3533, E10.3539, E10.3541, E10.3542, E10.3543, E10.3549, E10.3551, E10.3552, E10.3553, E10.3559, E10.359, E10.3591, E10.3592, E10.3593, E10.3599, E10.36, E10.37X1, E10.37X2, E10.37X3, E10.37X9, E10.39, E10.40, E10.41, E10.42, E10.43, E10.44, E10.49, E10.51, E10.52, E10.59, E10.610, E10.618, E10.620, E10.621, E10.622, E10.628, E10.630, E10.638, E10.641, E10.649, E10.65, E10.69, E10.8, E10.9, E11.00, E11.01, E11.10, E11.11, E11.21, E11.22, E11.29, E11.311, E11.319, E11.321, E11.3211, E11.3212, E11.3213, E11.3219, E11.329, E11.3291, E11.3292, E11.3293, E11.3299, E11.331, E11.3311, E11.3312, E11.3313, E11.3319, E11.339, E11.3391, E11.3392, E11.3393, E11.3399, E11.341, E11.3411, E11.3412, E11.3413, E11.3419, E11.349, E11.3491, E11.3492, E11.3493, E11.3499, E11.351, E11.3511, E11.3512, E11.3513, E11.3519, E11.3521, E11.3522, E11.3523, E11.3529, E11.3531, E11.3532, E11.3533, E11.3539, E11.3541, E11.3542, E11.3543, E11.3549, E11.3551, E11.3552, E11.3553, E11.3559, E11.359, E11.3591, E11.3592, E11.3593, E11.3599, E11.36, E11.37X1, E11.37X2, E11.37X3, E11.37X9, E11.39, E11.40, E11.41, E11.42, E11.43, E11.44, E11.49, E11.51, E11.52, E11.59, E11.610, E11.618, E11.620, E11.621, E11.622, E11.628, E11.630, E11.638, E11.641, E11.649, E11.65, E11.69, E11.8, E11.9, E13.00, E13.01, E13.10, E13.11, E13.21, E13.22, E13.29, E13.311, E13.319, E13.321, E13.3211, E13.3212, E13.3213, E13.3219, E13.329, E13.3291, E13.3292, E13.3293, E13.3299, E13.331, E13.3311, E13.3312, E13.3313, E13.3319, E13.339, E13.3391, E13.3392, E13.3393, E13.3399, E13.341, E13.3411, E13.3412, E13.3413, E13.3419, E13.349, E13.3491, E13.3492, E13.3493, E13.3499, E13.351, E13.3511, E13.3512, E13.3513, E13.3519, E13.3521, E13.3522, E13.3523, E13.3529, E13.3531, E13.3532, E13.3533, E13.3539, E13.3541, E13.3542, E13.3543, E13.3549, E13.3551, E13.3552, E13.3553, E13.3559, E13.359, E13.3591, E13.3592, E13.3593, E13.3599, E13.36, E13.39, E13.40, E13.41, E13.42, E13.43, E13.44, E13.49, E13.51, E13.52, E13.59, E13.610, E13.618, E13.620, E13.621, E13.622, E13.628, E13.630, E13.638, E13.641, E13.649, E13.65, E13.69, E13.8, E13.9 (any DX on the claim |
| Epilepsy | DX 345, 345.0, 345.00, 345.01, 345.1, 345.10, 345.11, 345.2, 345.3, 345.4, 345.40, 345.41, 345.5, 345.50, 345.51, 345.6, 345.60, 345.61, 345.7, 345.70, 345.71, 345.8, 345.80, 345.81, 345.9, 345.90, 345.91 (any DX on the claim) | DX G40.001, G40.009, G40.011, G40.019, G40.101, G40.109, G40.111, G40.119, G40.201, G40.209, G40.211, G40.219, G40.301, G40.309, G40.311, G40.319, G40.401, G40.409, G40.411, G40.419, G40.42, G40.501, G40.509, G40.801, G40.802, G40.803, G40.804, G40.811, G40.812, G40.813, G40.814, G40.821, G40.822, G40.823, G40.824, G40.833, G40.834, G40.89, G40.901, G40.909, G40.911, G40.919, G40.A01, G40.A09, G40.A11, G40.A19, G40.B01, G40.B09, G40.B11, G40.B19 (any DX on the claim) |
| Glaucoma | 362.85, 365.00, 365.01, 365.02, 365.03, 365.04, 365.10, 365.11, 365.12, 365.13, 365.15, 365.20, 365.21, 365.22, 365.23, 365.24, 365.31, 365.32, 365.41, 365.42, 365.43, 365.51, 365.52, 365.59, 365.60, 365.61, 365.62, 365.63, 365.64, 365.65, 365.81, 365.82, 365.83, 365.89, 365.9, 377.14 (ONLY principal DX on the claim) | H40.011, H40.012, H40.013, H40.019, H40.021, H40.022, H40.023, H40.029, H40.041, H40.042, H40.043, H40.049, H40.051, H40.052, H40.053, H40.059, H40.10X0, H40.10X1, H40.10X2, H40.10X3, H40.10X4, H40.1110, H40.1111, H40.1112, H40.1113, H40.1114, H40.1120, H40.1121, H40.1122, H40.1123, H40.1124, H40.1130, H40.1131, H40.1132, H40.1133, H40.1134, H40.1190, H40.1191, H40.1192, H40.1193, H40.1194, H40.11X0, H40.11X1, H40.11X2, H40.11X3, H40.11X4, H40.1210, H40.1211, H40.1212, H40.1213, H40.1214, H40.1220, H40.1221, H40.1222, H40.1223, H40.1224, H40.1230, H40.1231, H40.1232, H40.1233, H40.1234, H40.1290, H40.1291, H40.1292, H40.1293, H40.1294, H40.1310, H40.1311, H40.1312, H40.1313, H40.1314, H40.1320, H40.1321, H40.1322, H40.1323, H40.1324, H40.1330, H40.1331, H40.1332, H40.1333, H40.1334, H40.1390, H40.1391, H40.1392, H40.1393, H40.1394, H40.1410, H40.1411, H40.1412, H40.1413, H40.1414, H40.1420, H40.1421, H40.1422, H40.1423, H40.1424, H40.1430, H40.1431, H40.1432, H40.1433, H40.1434, H40.1490, H40.1491, H40.1492, H40.1493, H40.1494, H40.151, H40.152, H40.153, H40.159, H40.20X0, H40.20X1, H40.20X2, H40.20X3, H40.20X4, H40.211, H40.212, H40.213, H40.219, H40.2210, H40.2211, H40.2212, H40.2213, H40.2214, H40.2220, H40.2221, H40.2222, H40.2223, H40.2224, H40.2230, H40.2231, H40.2232, H40.2233, H40.2234, H40.2290, H40.2291, H40.2292, H40.2293, H40.2294, H40.231, H40.232, H40.233, H40.239, H40.241, H40.242, H40.243, H40.249, H40.30X0, H40.30X1, H40.30X2, H40.30X3, H40.30X4, H40.31X0, H40.31X1, H40.31X2, H40.31X3, H40.31X4, H40.32X0, H40.32X1, H40.32X2, H40.32X3, H40.32X4, H40.33X0, H40.33X1, H40.33X2, H40.33X3, H40.33X4, H40.40X0, H40.40X1, H40.40X2, H40.40X3, H40.40X4, H40.41X0, H40.41X1, H40.41X2, H40.41X3, H40.41X4, H40.42X0, H40.42X1, H40.42X2, H40.42X3, H40.42X4, H40.43X0, H40.43X1, H40.43X2, H40.43X3, H40.43X4, H40.50X0, H40.50X1, H40.50X2, H40.50X3, H40.50X4, H40.51X0, H40.51X1, H40.51X2, H40.51X3, H40.51X4, H40.52X0, H40.52X1, H40.52X2, H40.52X3, H40.52X4, H40.53X0, H40.53X1, H40.53X2, H40.53X3, H40.53X4, H40.60X0, H40.60X1, H40.60X2, H40.60X3, H40.60X4, H40.61X0, H40.61X1, H40.61X2, H40.61X3, H40.61X4, H40.62X0, H40.62X1, H40.62X2, H40.62X3, H40.62X4, H40.63X0, H40.63X1, H40.63X2, H40.63X3, H40.63X4, H40.811, H40.812, H40.813, H40.819, H40.821, H40.822, H40.823, H40.829, H40.831, H40.832, H40.833, H40.839, H40.89, H40.9, H42, H44.511, H44.512, H44.513, H44.519, H47.231, H47.232, H47.233, H47.239, Q15.0 (any DX on the claim) |
| Hypertension | 362.11, 401.0, 401.1, 401.9, 402.00, 402.01, 402.10, 402.11, 402.90, 402.91, 403.00, 403.01, 403.10, 403.11, 403.90, 403.91, 404.00, 404.01, 404.02, 404.03, 404.10, 404.11, 404.12, 404.13, 404.90, 404.91, 404.92, 404.93, 405.01, 405.09, 405.11, 405.19, 405.91, 405.99, 437.2 (any DX on the claim) | H35.031, H35.032, H35.033, H35.039, I10, I11.0, I11.9, I12.0, I12.9, I13.0, I13.10, I13.11, I13.2, I15.0, I15.1, I15.2, I15.8, I15.9, I67.4, N26.2 (any DX on the claim) |
| Ischemic heart disease | 410.00, 410.01, 410.02, 410.10, 410.11, 410.12, 410.20, 410.21, 410.22, 410.30, 410.31, 410.32, 410.40, 410.41, 410.42, 410.50, 410.51, 410.52, 410.60, 410.61, 410.62, 410.70, 410.71, 410.72, 410.80, 410.81, 410.82, 410.90, 410.91, 410.92, 411.0, 411.1, 411.81, 411.89, 412, 413.0, 413.1, 413.9, 414.00, 414.01, 414.02, 414.03, 414.04, 414.05, 414.06, 414.07, 414.12, 414.2, 414.3, 414.4, 414.8, 414.9 (any DX on the claim) | I20.0, I20.1, I20.2, I20.8, I24.0, I24.1, I24.8, I25.10, I25.110, I25.111, I25.112, I25.118, I25.119, I25.3, I25.41, I25.42, I25.5, I25.6, I25.700, I25.701, I25.702, I25.708, I25.710, I25.711, I25.712, I25.718, I25.719, I25.720, I25.721, I25.722, I25.728, I25.729, I25.730, I25.731, I25.732, I25.738, I25.739, I25.750, I25.751, I25.752, I25.758, I25.759, I25.760, I25.761, I25.762, I25.768, I25.769, I25.790, I25.791, I25.792, I25.798, I25.799, I25.810, I25.811, I25.812, I25.82, I25.83, I25.84, I25.89, I25.9 (any DX on the claim) |
| Migraine and other chronic headache | DX 339, 339.0, 339.00, 339.01, 339.02, 339.03, 339.04, 339.05, 339.09, 339.1, 339.10, 339.11, 339.12, 339.2, 339.20, 339.21, 339.22, 339.3, 339.4, 339.41, 339.42, 339.43, 339.44, 339.8, 339.81, 339.82, 339.83, 339.84, 339.85, 339.89, 346, 346.0, 346.00, 346.01, 346.02, 346.03, 346.1, 346.10, 346.11, 346.12, 346.13, 346.2, 346.20, 346.21, 346.22, 346.23, 346.3, 346.30, 346.31, 346.32, 346.33, 346.4, 346.40, 346.41, 346.42, 346.43, 346.5, 346.50, 346.51, 346.52, 346.53, 346.6, 346.60, 346.61, 346.62, 346.63, 346.7, 346.70, 346.71, 346.72, 346.73, 346.8, 346.80, 346.81, 346.82, 346.83, 346.9, 346.90, 346.91, 346.92, 346.93 (any DX on the claim) | DX G43.001, G43.009, G43.011, G43.019, G43.101, G43.109, G43.111, G43.119, G43.401, G43.409, G43.411, G43.419, G43.501, G43.509, G43.511, G43.519, G43.601, G43.609, G43.611, G43.619, G43.701, G43.709, G43.711, G43.719, G43.A0, G43.B0, G43.C0, G43.D0, G43.A1, G43.B1, G43.C1, G43.D1, G43.801, G43.809, G43.811, G43.819, G43.821, G43.829, G43.831, G43.839, G43.901, G43.909, G43.911, G43.919, G44.001, G44.009, G44.011, G44.019, G44.021, G44.029, G44.031, G44.039, G44.041, G44.049, G44.051, G44.059, G44.091, G44.099, G44.1, G44.201, G44.209, G44.211, G44.219, G44.221, G44.229, G44.301, G44.309, G44.311, G44.319, G44.321, G44.329, G44.40, G44.41, G44.51, G44.52, G44.53, G44.59, G44.81, G44.82, G44.83, G44.84, G44.85, G44.86, G44.89 (any DX on the claim) |
| Mobility impairments | DX 334.1, 342.00, 342.01, 342.02, 342.10, 342.11, 342.12, 342.80, 342.81, 342.82, 342.90, 342.91, 342.92, 344, 344.0, 344.00, 344.01, 344.02, 344.03, 344.04, 344.09, 344.1, 344.2, 344.3, 344.30, 344.31, 344.32, 344.4, 344.40, 344.41, 344.42, 344.5, 344.6, 344.60, 344.61, 344.8, 344.81, 344.89, 344.9, 438.20, 438.21, 438.22, 438.30, 438.31, 438.32, 438.40, 438.41, 438.42, 438.50, 438.51, 438.52, 438.53 (any DX on the claim) | DX G04.1, G11.4, G81.00, G81.01, G81.02, G81.03, G81.04, G81.10, G81.11, G81.12, G81.13, G81.14, G81.90, G81.91, G81.92, G81.93, G81.94, G82.20, G82.21, G82.22, G82.50, G82.51, G82.52, G82.53, G82.54, G83.0, G83.10, G83.11, G83.12, G83.13, G83.14, G83.20, G83.21, G83.22, G83.23, G83.24, G83.30, G83.31, G83.32, G83.33, G83.34, G83.4, G83.5, G83.81, G83.82, G83.83, G83.84, G83.89, G83.9, I69.031, I69.032, I69.033, I69.034, I69.039, I69.041, I69.042, I69.043, I69.044, I69.049, I69.051, I69.052, I69.053, I69.054, I69.059, I69.061, I69.062, I69.063, I69.064, I69.065, I69.069, I69.131, I69.132, I69.133, I69.134, I69.139, I69.141, I69.142, I69.143, I69.144, I69.149, I69.151, I69.152, I69.153, I69.154, I69.159, I69.161, I69.162, I69.163, I69.164, I69.165, I69.169, I69.231, I69.232, I69.233, I69.234, I69.239, I69.241, I69.242, I69.243, I69.244, I69.249, I69.251, I69.252, I69.253, I69.254, I69.259, I69.261, I69.262, I69.263, I69.264, I69.265, I69.269, I69.331, I69.332, I69.333, I69.334, I69.339, I69.341, I69.342, I69.343, I69.344, I69.349, I69.351, I69.352, I69.353, I69.354, I69.359, I69.361, I69.362, I69.363, I69.364, I69.365, I69.369, I69.831, I69.832, I69.833, I69.834, I69.839, I69.841, I69.842, I69.843, I69.844, I69.849, I69.851, I69.852, I69.853, I69.854, I69.859, I69.861, I69.862, I69.863, I69.864, I69.865, I69.869, I69.931, I69.932, I69.933, I69.934, I69.939, I69.941, I69.942, I69.943, I69.944, I69.949, I69.951, I69.952, I69.953, I69.954, I69.959, I69.961, I69.962, I69.963, I69.964, I69.965, I69.969 (any DX on the claim) |
| Peripheral vascular disease | DX 440.0, 440.1, 440.2, 440.20, 440.21, 440.22, 440.23, 440.29, 440.4, 443.8, 443.81, 443.82, 443.89, 443.9 (any DX on the claim) | DX E08.51, E08.52, E09.51, E09.52, E10.51, E10.52, E11.51, E11.52, E13.51, E13.52, I70.0, I70.1, I70.201, I70.202, I70.203, I70.208, I70.209, I70.211, I70.212, I70.213, I70.218, I70.219, I70.221, I70.222, I70.223, I70.228, I70.229, I70.231, I70.232, I70.233, I70.234, I70.235, I70.238, I70.239, I70.241, I70.242, I70.243, I70.244, I70.245, I70.248, I70.249, I70.25, I70.291, I70.292, I70.293, I70.298, I70.299, I70.92, I73.81, I73.89, I73.9, I79.1, I79.8 (any DX on the claim) |
| Rheumatoid/Osteoarthritis | 714.0, 714.1, 714.2, 714.30, 714.31, 714.32, 714.33, 715.00, 715.04, 715.09, 715.10, 715.11, 715.12, 715.13, 715.14, 715.15, 715.16, 715.17, 715.18, 715.20, 715.21, 715.22, 715.23, 715.24, 715.25, 715.26, 715.27, 715.28, 715.30, 715.31, 715.32, 715.33, 715.34, 715.35, 715.36, 715.37, 715.38, 715.80, 715.89, 715.90, 715.91, 715.92, 715.93, 715.94, 715.95, 715.96, 715.97, 715.98, 720.0, 721.0, 721.1, 721.2, 721.3, 721.90, 721.91 (any DX on the claim) | M05.00, M05.011, M05.012, M05.019, M05.021, M05.022, M05.029, M05.031, M05.032, M05.039, M05.041, M05.042, M05.049, M05.051, M05.052, M05.059, M05.061, M05.062, M05.069, M05.071, M05.072, M05.079, M05.09, M05.20, M05.211, M05.212, M05.219, M05.221, M05.222, M05.229, M05.231, M05.232, M05.239, M05.241, M05.242, M05.249, M05.251, M05.252, M05.259, M05.261, M05.262, M05.269, M05.271, M05.272, M05.279, M05.29, M05.30, M05.311, M05.312, M05.319, M05.321, M05.322, M05.329, M05.331, M05.332, M05.339, M05.341, M05.342, M05.349, M05.351, M05.352, M05.359, M05.361, M05.362, M05.369, M05.371, M05.372, M05.379, M05.39, M05.40, M05.411, M05.412, M05.419, M05.421, M05.422, M05.429, M05.431, M05.432, M05.439, M05.441, M05.442, M05.449, M05.451, M05.452, M05.459, M05.461, M05.462, M05.469, M05.471, M05.472, M05.479, M05.49, M05.50, M05.511, M05.512, M05.519, M05.521, M05.522, M05.529, M05.531, M05.532, M05.539, M05.541, M05.542, M05.549, M05.551, M05.552, M05.559, M05.561, M05.562, M05.569, M05.571, M05.572, M05.579, M05.59, M05.60, M05.611, M05.612, M05.619, M05.621, M05.622, M05.629, M05.631, M05.632, M05.639, M05.641, M05.642, M05.649, M05.651, M05.652, M05.659, M05.661, M05.662, M05.669, M05.671, M05.672, M05.679, M05.69, M05.70, M05.711, M05.712, M05.719, M05.721, M05.722, M05.729, M05.731, M05.732, M05.739, M05.741, M05.742, M05.749, M05.751, M05.752, M05.759, M05.761, M05.762, M05.769, M05.771, M05.772, M05.779, M05.79, M05.7A, M05.80, M05.811, M05.812, M05.819, M05.821, M05.822, M05.829, M05.831, M05.832, M05.839, M05.841, M05.842, M05.849, M05.851, M05.852, M05.859, M05.861, M05.862, M05.869, M05.871, M05.872, M05.879, M05.89, M05.8A, M05.9, M06.00, M06.011, M06.012, M06.019, M06.021, M06.022, M06.029, M06.031, M06.032, M06.039, M06.041, M06.042, M06.049, M06.051, M06.052, M06.059, M06.061, M06.062, M06.069, M06.071, M06.072, M06.079, M06.08, M06.09, M06.0A, M06.1, M06.20, M06.211, M06.212, M06.219, M06.221, M06.222, M06.229, M06.231, M06.232, M06.239, M06.241, M06.242, M06.249, M06.251, M06.252, M06.259, M06.261, M06.262, M06.269, M06.271, M06.272, M06.279, M06.28, M06.29, M06.30, M06.311, M06.312, M06.319, M06.321, M06.322, M06.329, M06.331, M06.332, M06.339, M06.341, M06.342, M06.349, M06.351, M06.352, M06.359, M06.361, M06.362, M06.369, M06.371, M06.372, M06.379, M06.38, M06.39, M06.80, M06.811, M06.812, M06.819, M06.821, M06.822, M06.829, M06.831, M06.832, M06.839, M06.841, M06.842, M06.849, M06.851, M06.852, M06.859, M06.861, M06.862, M06.869, M06.871, M06.872, M06.879, M06.88, M06.89, M06.8A, M06.9, M08.00, M08.011, M08.012, M08.019, M08.021, M08.022, M08.029, M08.031, M08.032, M08.039, M08.041, M08.042, M08.049, M08.051, M08.052, M08.059, M08.061, M08.062, M08.069, M08.071, M08.072, M08.079, M08.08, M08.09, M08.0A, M08.1, M08.20, M08.211, M08.212, M08.219, M08.221, M08.222, M08.229, M08.231, M08.232, M08.239, M08.241, M08.242, M08.249, M08.251, M08.252, M08.259, M08.261, M08.262, M08.269, M08.271, M08.272, M08.279, M08.28, M08.29, M08.2A, M08.3, M08.40, M08.411, M08.412, M08.419, M08.421, M08.422, M08.429, M08.431, M08.432, M08.439, M08.441, M08.442, M08.449, M08.451, M08.452, M08.459, M08.461, M08.462, M08.469, M08.471, M08.472, M08.479, M08.48, M08.4A, M08.80, M08.811, M08.812, M08.819, M08.821, M08.822, M08.829, M08.831, M08.832, M08.839, M08.841, M08.842, M08.849, M08.851, M08.852, M08.859, M08.861, M08.862, M08.869, M08.871, M08.872, M08.879, M08.88, M08.89, M08.90, M08.911, M08.912, M08.919, M08.921, M08.922, M08.929, M08.931, M08.932, M08.939, M08.941, M08.942, M08.949, M08.951, M08.952, M08.959, M08.961, M08.962, M08.969, M08.971, M08.972, M08.979, M08.98, M08.99, M08.9A, M15.0, M15.1, M15.2, M15.3, M15.4, M15.8, M15.9, M16.0, M16.10, M16.11, M16.12, M16.2, M16.30, M16.31, M16.32, M16.4, M16.50, M16.51, M16.52, M16.6, M16.7, M16.9, M17.0, M17.10, M17.11, M17.12, M17.2, M17.30, M17.31, M17.32, M17.4, M17.5, M17.9, M18.0, M18.10, M18.11, M18.12, M18.2, M18.30, M18.31, M18.32, M18.4, M18.50, M18.51, M18.52, M18.9, M19.011, M19.012, M19.019, M19.021, M19.022, M19.029, M19.031, M19.032, M19.039, M19.041, M19.042, M19.049, M19.071, M19.072, M19.079, M19.09, M19.111, M19.112, M19.119, M19.121, M19.122, M19.129, M19.131, M19.132, M19.139, M19.141, M19.142, M19.149, M19.171, M19.172, M19.179, M19.19, M19.211, M19.212, M19.219, M19.221, M19.222, M19.229, M19.231, M19.232, M19.239, M19.241, M19.242, M19.249, M19.271, M19.272, M19.279, M19.29, M19.90, M19.91, M19.92, M19.93, M45.0, M45.1, M45.2, M45.3, M45.4, M45.5, M45.6, M45.7, M45.8, M45.9, M47.011, M47.012, M47.013, M47.014, M47.015, M47.016, M47.019, M47.021, M47.022, M47.029, M47.10, M47.11, M47.12, M47.13, M47.20, M47.21, M47.22, M47.23, M47.24, M47.25, M47.26, M47.27, M47.28, M47.811, M47.812, M47.813, M47.814, M47.815, M47.816, M47.817, M47.818, M47.819, M47.891, M47.892, M47.893, M47.894, M47.895, M47.896, M47.897, M47.898, M47.899, M47.9, M48.8X1, M48.8X2, M48.8X3, M48.8X4, M48.8X5, M48.8X6, M48.8X7, M48.8X8, M48.8X9 (any DX on the claim) |
| Schizophrenia and other psychotic disorders | DX 293.81, 293.82, 295.00, 295.01, 295.02, 295.03, 295.04, 295.05, 295.10, 295.11, 295.12, 295.13, 295.14, 295.15, 295.20, 295.21, 295.22, 295.23, 295.24, 295.25, 295.30, 295.31, 295.32, 295.33, 295.34, 295.35, 295.40, 295.41, 295.42, 295.43, 295.44, 295.45, 295.50, 295.51, 295.52, 295.53, 295.54, 295.55, 295.60, 295.61, 295.62, 295.63, 295.64, 295.65, 295.70, 295.71, 295.72, 295.73, 295.74, 295.75, 295.80, 295.81, 295.82, 295.83, 295.84, 295.85, 295.90, 295.91, 295.92, 295.93, 295.94, 295.95, 297.0, 297.1, 297.2, 297.3, 297.8, 297.9, 298.0, 298.1, 298.2, 298.3, 298.4, 298.8, 298.9 (any DX on the claim) | DX F06.0, F06.2, F20.0, F20.1, F20.2, F20.3, F20.5, F20.81, F20.89, F20.9, F21, F22, F23, F24, F25.0, F25.1, F25.8, F25.9, F28, F29, F32.3, F33.3, F44.89 (any DX on the claim) |
| Sensory - blindness and visual impairment | DX 369, 369.0, 369.00, 369.01, 369.02, 369.03, 369.04, 369.05, 369.06, 369.07, 369.08, 369.1, 369.10, 369.11, 369.12, 369.13, 369.14, 369.15, 369.16, 369.17, 369.18, 369.2, 369.20, 369.21, 369.22, 369.23, 369.24, 369.25, 369.3, 369.4 (any DX on the claim) | DX H54.0, H54.0X33, H54.0X34, H54.0X35, H54.0X43, H54.0X44, H54.0X45, H54.0X53, H54.0X54, H54.0X55, H54.10, H54.11, H54.1131, H54.1132, H54.1141, H54.1142, H54.1151, H54.1152, H54.12, H54.1213, H54.1214, H54.1215, H54.1223, H54.1224, H54.1225, H54.2, H54.2X11, H54.2X12, H54.2X21, H54.2X22, H54.3, H54.8 (any DX on the claim) |
| Sensory - deafness and hearing impairment | DX 389, 389.1, 389.10, 389.11, 389.12, 389.13, 389.14, 389.15, 389.16, 389.17, 389.18, 389.2, 389.20, 389.21, 389.22, 389.7, 389.8, 389.9 (any DX on the claim) | DX H90.3, H90.41, H90.42, H90.5, H90.6, H90.71, H90.72, H90.8, H90.A21, H90.A22, H90.A31, H90.A32, H91.01, H91.02, H91.03, H91.09, H91.3, H91.8X1, H91.8X2, H91.8X3, H91.8X9, H91.90, H91.91, H91.92, H91.93 (any DX on the claim) |
| Stroke or trans ischemic attack |  | G45.0, G45.1, G45.2, G45.3, G45.8, G45.9, G46.0, G46.1, G46.2, G46.3, G46.4, G46.5, G46.6, G46.7, G46.8, G97.31, G97.32, I60.00, I60.01, I60.02, I60.10, I60.11, I60.12, I60.2, I60.20, I60.21, I60.22, I60.30, I60.31, I60.32, I60.4, I60.50, I60.51, I60.52, I60.6, I60.7, I60.8, I60.9, I61.0, I61.1, I61.2, I61.3, I61.4, I61.5, I61.6, I61.8, I61.9, I62.00, I62.01, I62.02, I62.9, I63.00, I63.011, I63.012, I63.013, I63.019, I63.02, I63.031, I63.032, I63.033, I63.039, I63.09, I63.10, I63.111, I63.112, I63.113, I63.119, I63.12, I63.131, I63.132, I63.133, I63.139, I63.19, I63.20, I63.211, I63.212, I63.213, I63.219, I63.22, I63.231, I63.232, I63.233, I63.239, I63.29, I63.30, I63.311, I63.312, I63.313, I63.319, I63.321, I63.322, I63.323, I63.329, I63.331, I63.332, I63.333, I63.339, I63.341, I63.342, I63.343, I63.349, I63.39, I63.40, I63.411, I63.412, I63.413, I63.419, I63.421, I63.422, I63.423, I63.429, I63.431, I63.432, I63.433, I63.439, I63.441, I63.442, I63.443, I63.449, I63.49, I63.50, I63.511, I63.512, I63.513, I63.519, I63.521, I63.522, I63.523, I63.529, I63.531, I63.532, I63.533, I63.539, I63.541, I63.542, I63.543, I63.549, I63.59, I63.6, I63.8, I63.81, I63.89, I63.9, I67.841, I67.848, I67.89, I97.810, I97.811, I97.820, I97.821 (any DX on the claim) EXCLUSION: If any of the qualifying claims have any of the following codes in any DX position then EXCLUDE: S06.340A, S06.341A, S06.342A, S06.343A, S06.344A, S06.345A, S06.346A, S06.347A, S06.348A, S06.34AA, S06.349A, S06.350A, S06.351A, S06.352A, S06.353A, S06.354A, S06.355A, S06.356A, S06.357A, S06.358A, S06.35AA, S06.359A, S06.360A, S06.361A, S06.362A, S06.363A, S06.364A, S06.365A, S06.366A, S06.367A, S06.368A, S06.36AA, S06.369A, S06.370A, S06.371A, S06.372A, S06.373A, S06.374A, S06.375A, S06.376A, S06.377A, S06.378A, S06.37AA, S06.379A, S06.380A, S06.381A, S06.382A, S06.383A, S06.384A, S06.385A, S06.386A, S06.387A, S06.388A, S06.38AA, S06.389A, S06.5X0A, S06.5X1A, S06.5X2A, S06.5X3A, S06.5X4A, S06.5X5A, S06.5X6A, S06.5X7A, S06.5X8A, S06.5XAA, S06.5X9A, S06.6X0A, S06.6X1A, S06.6X2A, S06.6X3A, S06.6X4A, S06.6X5A, S06.6X6A, S06.6X7A, S06.6X8A, S06.6XAA, S06.6X9A, S06.810A, S06.811A, S06.812A, S06.813A, S06.814A, S06.815A, S06.816A, S06.817A, S06.818A, S06.81AA, S06.819A, S06.820A, S06.821A, S06.822A, S06.823A, S06.824A, S06.825A, S06.826A, S06.827A, S06.828A, S06.82AA, S06.829A, S06.890A, S06.891A, S06.892A, S06.893A, S06.894A, S06.895A, S06.896A, S06.897A, S06.898A, S06.89AA, S06.899A, S06.9X0A, S06.9X1A, S06.9X2A, S06.9X3A, S06.9X4A, S06.9X5A, S06.9X6A, S06.9X7A, S06.9X8A, S06.9XAA, S06.9X9A, S06.A0XA, S06.A1XA |
| ICD = International Classification of Disease | | |
